# Supplementary material for: Adaptive force-position-speed collaborative process planning and roughness prediction for robotic polishing
Source: PLoS One. 2025 Sep 3;20(9):e0330979. doi: 10.1371/journal.pone.0330979 (PMC12407474; doi:10.1371/journal.pone.0330979)
Supplement: S1 File — (PDF) [file pone.0330979.s001.pdf]

Adaptive Force-Position-Speed Collaborative Process Planning and Roughness Prediction for Robotic Polishing

This study proposes an adaptive robotic polishing force-position-speed collaborative process planning framework. The improved dung beetle optimization algorithm, back propagation neural network, finite element analysis and response surface method provide a strong guarantee for the selection of robotic polishing process parameters. The adaptive impedance control strategy is implemented to enhance force control, and PD iteration and RBF neural network are used to ensure stable contact force and accuracy.

The source code and supporting materials related to this study are uploaded and shared: https://doi.org/10.6084/m9.figshare.29917520

****

The following is a description of the supporting materials.

It contains a total of 6 parts：

1. Improved DBO algorithm

This part is mainly about the improvement of DBO algorithm. The author has tried to solve various engineering problems using the improved DBO algorithm and achieved good results. This paper does not provide a detailed description of the strategy for further improvement of DBO algorithm. Please refer to the author's other papers.

The document shares the improved DBO algorithm for solving robot inverse kinematics, four engineering problems, and optimization solutions related to this study. Its superiority compared to multiple algorithms is evaluated.

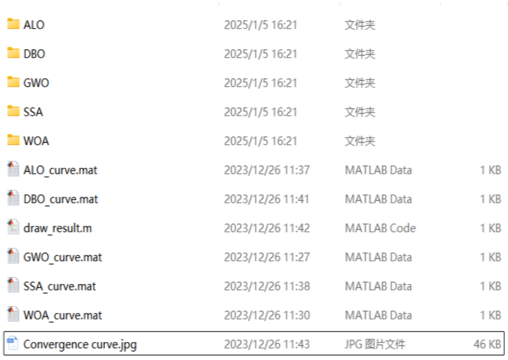

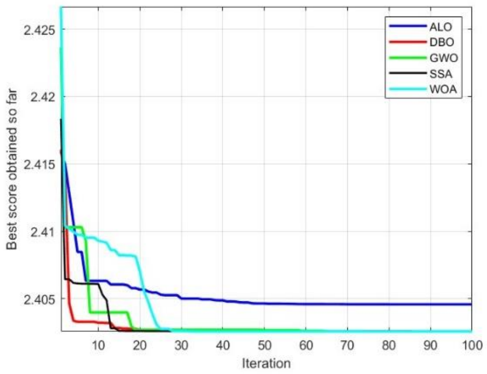

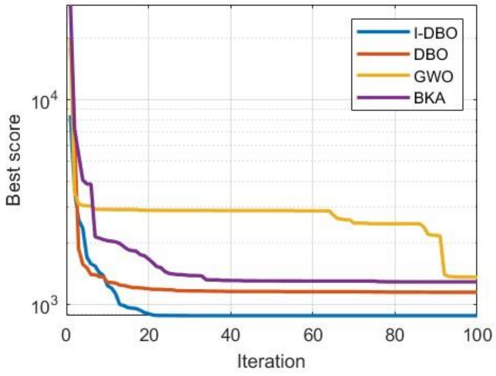

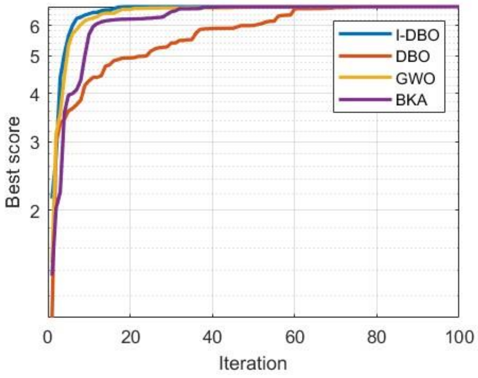

2. Curvature adaptive trajectory

This part of the paper presents the curvature adaptive interpolation algorithm. A surface example is solved and the results are obtained.

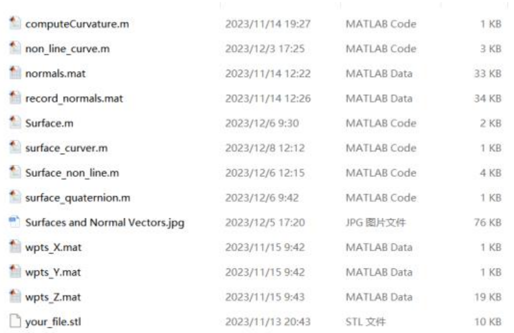

computeCurvature.m	2023/11/14 19:27	MATLAB Code	1 KB
non_line_curve.m	2023/12/3 17:25	MATLAB Code	3 KB
normals.mat	2023/11/14 12:22	MATLAB Data	33 KB
record_normals.mat	2023/11/14 12:26	MATLAB Data	34 KB
Surface.m	2023/12/6 9:30	MATLAB Code	2 KB
surface_curver.m	2023/12/8 12:12	MATLAB Code	1 KB
Surface_non_line.m	2023/12/6 12:15	MATLAB Code	4 KB
surface_quaternion.m	2023/12/6 9:42	MATLAB Code	1 KB
Surfaces and Normal Vectors.jpg	2023/12/5 17:20	JPG 图片文件	76 KB
wpts_X.mat	2023/11/15 9:42	MATLAB Data	1 KB
wpts_Y.mat	2023/11/15 9:42	MATLAB Data	1 KB
wpts_Z.mat	2023/11/15 9:43	MATLAB Data	19 KB
your_file.stl	2023/11/13 20:43	STL 文件	10 KB

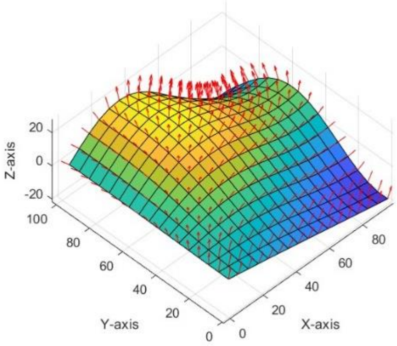

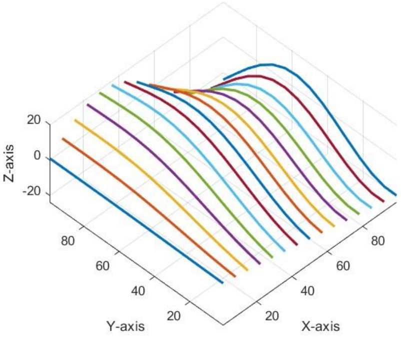

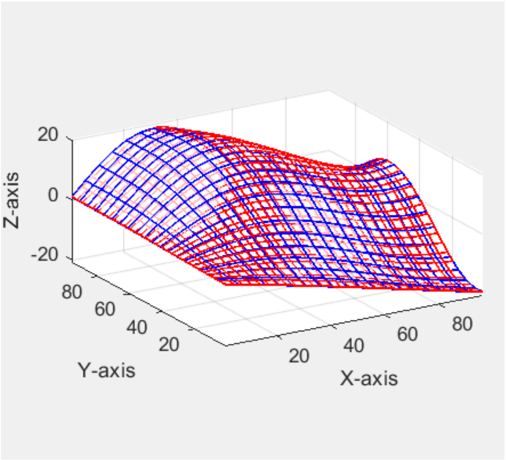

3. Finite Element Analysis (FEA)

The source files of the finite element analysis robot and polishing require 58.9G of storage, so only the results are uploaded.

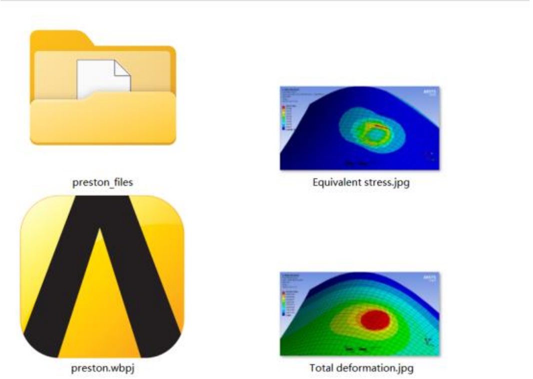

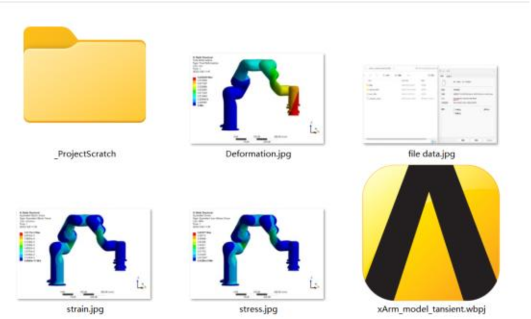

4. Adaptive impedance control

This document is the Matlab simulation and control results of the robot's adaptive impedance control, providing a theoretical basis for controlling robot polishing. Includes impedance control, dynamic parameters, LuGre friction estimation, etc.

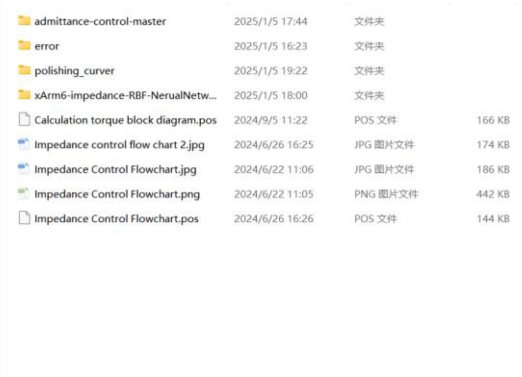

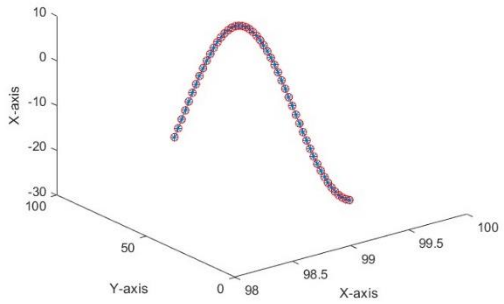

$$\dot{z} = v - \sigma_0 \frac{|v|}{g(v)} z, \qquad T_f = \sigma_0 z + \sigma_1 \dot{z} + \sigma_2 v.$$

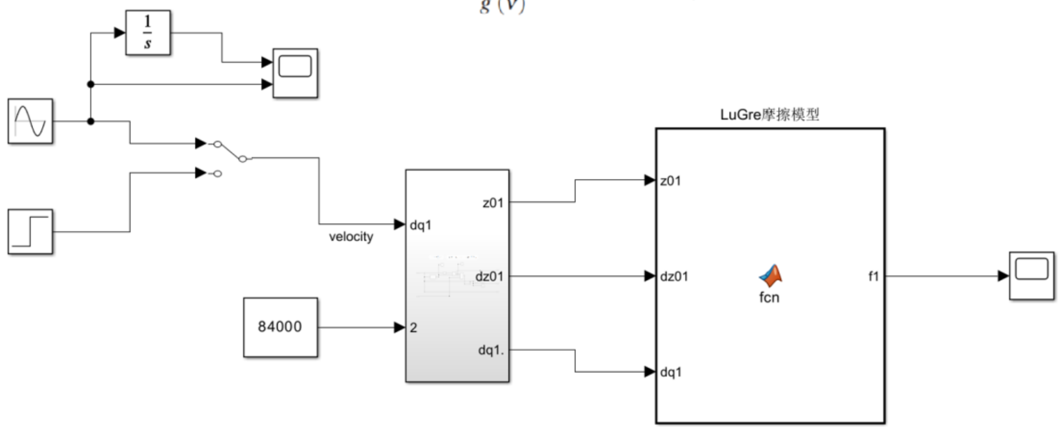

$$g(v) = F_c + (F_s - F_c)\exp^{-(|v/v_s|)}.$$

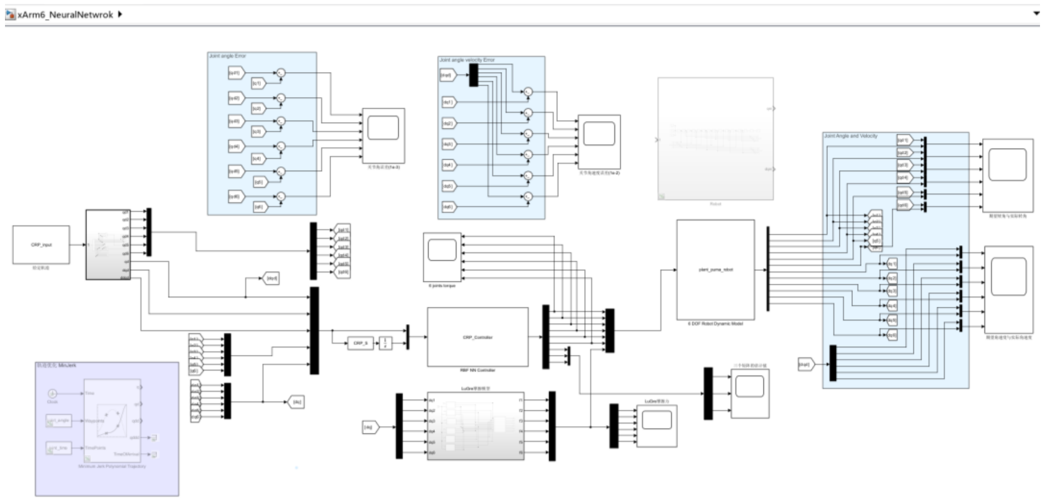

5. DBO-BPNN roughness prediction+RSM

This section includes the source code and results of BPNN model and response surface analysis.

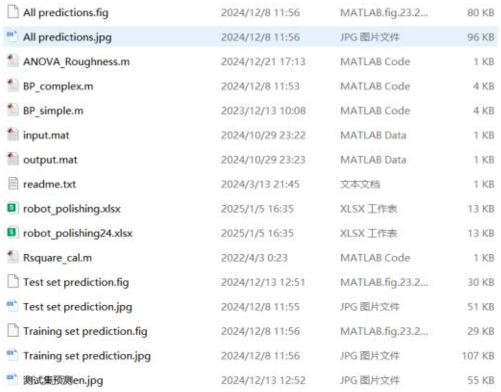

All predictions.fig	2024/12/8 11:56	MATLAB.fig.23.2...	80 KB
All predictions.jpg	2024/12/8 11:56	JPG 图片文件	96 KB
ANOVA_Roughness.m	2024/12/21 17:13	MATLAB Code	1 KB
BP_complex.m	2024/12/8 11:53	MATLAB Code	4 KB
BP_simple.m	2023/12/13 10:08	MATLAB Code	4 KB
input.mat	2024/10/29 23:22	MATLAB Data	1 KB
output.mat	2024/10/29 23:23	MATLAB Data	1 KB
readme.txt	2024/3/13 21:45	文本文档	1 KB
robot_polishing.xlsx	2025/1/5 16:35	XLSX 工作表	13 KB
robot_polishing24.xlsx	2025/1/5 16:35	XLSX 工作表	13 KB
Rsquare_cal.m	2022/4/3 0:23	MATLAB Code	1 KB
Test set prediction.fig	2024/12/13 12:51	MATLAB.fig.23.2...	30 KB
Test set prediction.jpg	2024/12/8 11:55	JPG 图片文件	51 KB
Training set prediction.fig	2024/12/8 11:56	MATLAB.fig.23.2...	29 KB
Training set prediction.jpg	2024/12/8 11:56	JPG 图片文件	107 KB
测试集预测en.jpg	2024/12/13 12:52	JPG 图片文件	55 KB

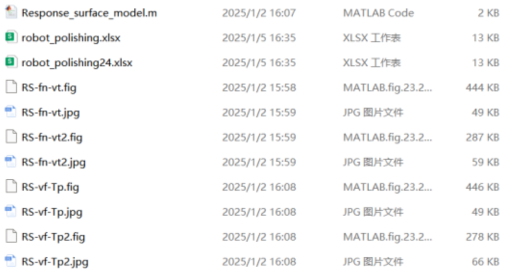

Response_surface_model.m	2025/1/2 16:07	MATLAB Code	2 KB
robot_polishing.xlsx	2025/1/5 16:35	XLSX 工作表	13 KB
robot_polishing24.xlsx	2025/1/5 16:35	XLSX 工作表	13 KB
RS-fn-vt.fig	2025/1/2 15:58	MATLAB.fig.23.2...	444 KB
RS-fn-vt.jpg	2025/1/2 15:59	JPG 图片文件	49 KB
RS-fn-vt2.fig	2025/1/2 15:59	MATLAB.fig.23.2...	287 KB
RS-fn-vt2.jpg	2025/1/2 15:59	JPG 图片文件	59 KB
RS-vf-Tp.fig	2025/1/2 16:08	MATLAB.fig.23.2...	446 KB
RS-vf-Tp.jpg	2025/1/2 16:08	JPG 图片文件	49 KB
RS-vf-Tp2.fig	2025/1/2 16:08	MATLAB.fig.23.2...	278 KB
RS-vf-Tp2.jpg	2025/1/2 16:08	JPG 图片文件	66 KB

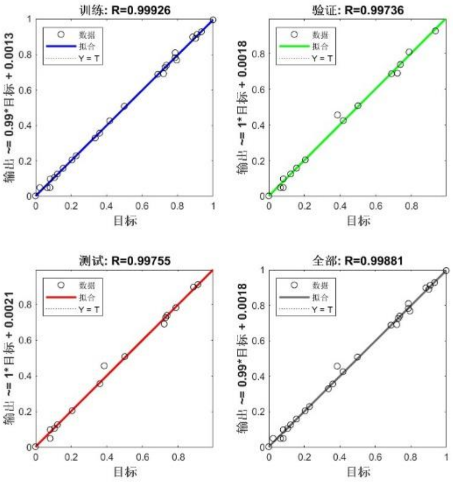

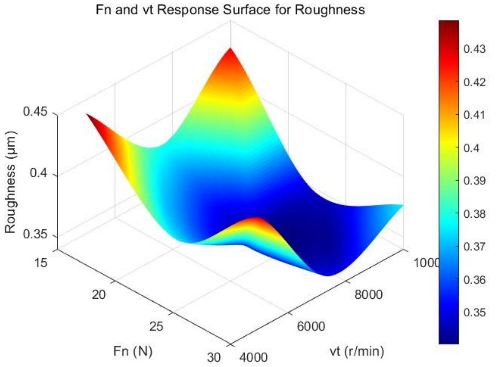

6. Robot polishing experiments

This section presents the scenario of the robotic polishing experiment and the obtained results.

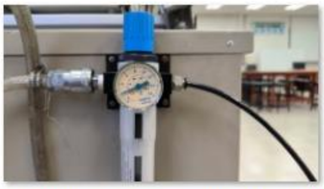

Air pressure speed regulation.jpg

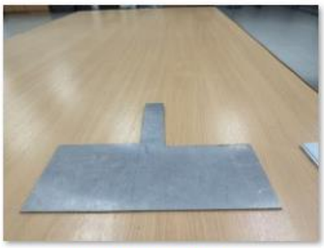

Aluminum alloy plate.jpg

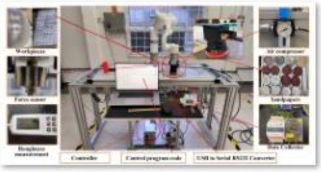

Experimental Platform.jpg

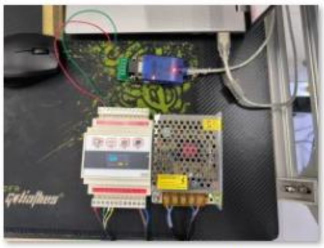

Force Collector.jpg

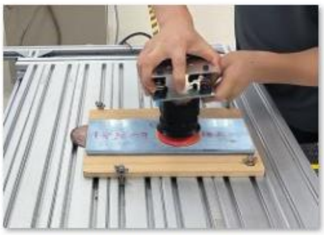

Manual polishing surface.jpg

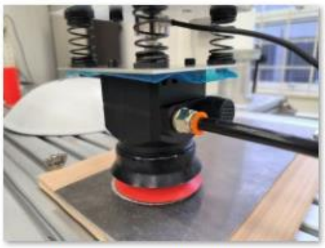

Polishing tools and force sensors.jpg

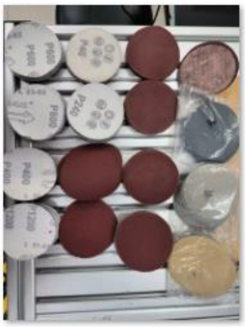

Sandpaper Type.jpg

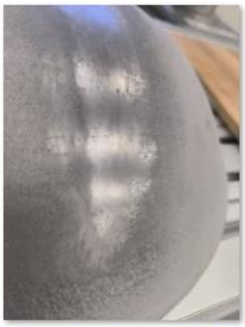

Surface polishing effect.jpg

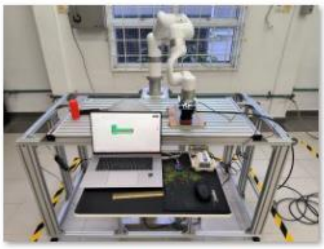

Test bench.jpg

1.png	2024/12/31 10:26	PNG 图片文件	2,699 KB
1.xlsx	2024/12/31 10:23	XLSX 工作表	241 KB
2.png	2024/12/31 10:26	PNG 图片文件	2,699 KB
2.xlsx	2024/12/31 10:24	XLSX 工作表	141 KB
3.png	2024/12/31 10:26	PNG 图片文件	2,699 KB
3.xlsx	2024/12/31 10:25	XLSX 工作表	56 KB
11.xlsx	2025/1/3 17:02	XLSX 工作表	325 KB
22.xlsx	2025/1/3 17:14	XLSX 工作表	203 KB
Force Control-Original.fig	2025/1/3 18:54	MATLAB.fig.23.2...	47 KB
Force Control-Original.jpg	2025/1/3 18:54	JPG 图片文件	71 KB
Force Control-Original1.jpg	2025/1/3 18:56	JPG 图片文件	146 KB
Force Control-Proposed.fig	2025/1/3 18:53	MATLAB.fig.23.2...	62 KB
Force Control-Proposed.jpg	2025/1/3 18:53	JPG 图片文件	67 KB
Force Control-Proposed1.jpg	2025/1/3 18:55	JPG 图片文件	139 KB
plot_force.m	2025/1/3 18:53	MATLAB Code	1 KB

Gambar	2025/1/5 18:18	文件夹	
Test roughness	2025/1/5 16:23	文件夹	
plot_roughness.m	2025/1/1 20:18	MATLAB Code	1 KB
rgns.fig	2025/1/1 18:50	MATLAB.fig.23.2...	503 KB
rgns.jpg	2025/1/1 18:49	JPG 图片文件	112 KB
Roughness measurement.mp4	2025/1/5 18:20	mp4 - MPEG-4 ...	4,640 KB
Roughness measuring instrument.jpg	2024/12/31 11:18	JPG 图片文件	260 KB
S1-A.xlsx	2024/11/1 11:27	XLSX 工作表	569 KB
S1-B.xlsx	2024/11/1 11:27	XLSX 工作表	575 KB
S1-C.xlsx	2024/11/1 11:27	XLSX 工作表	573 KB
S2-A.xlsx	2024/11/1 11:27	XLSX 工作表	549 KB
S2-B.xlsx	2024/11/1 11:27	XLSX 工作表	549 KB
S2-C.xlsx	2024/11/1 11:30	XLSX 工作表	550 KB
S3-A.xlsx	2024/11/1 11:30	XLSX 工作表	550 KB
S3-B.xlsx	2024/11/1 11:30	XLSX 工作表	553 KB
S3-C.xlsx	2024/11/1 11:30	XLSX 工作表	553 KB

Convex surface profile data extraction	2025/1/5 18:24	文件夹	
python-Test.py.tar.gz	2024/6/11 11:36	Bandizip.gz	2 KB
python-Test_1.py.tar.gz	2024/6/11 11:36	Bandizip.gz	2 KB
python-TEST_LEARN_1_CONTINUOUS.py.tar.gz	2024/6/11 11:36	Bandizip.gz	2 KB
python-TEST_LEARN_3_ARCLINEAR.py.tar.gz	2024/6/11 11:36	Bandizip.gz	2 KB
Polishing_P1.py	2025/1/5 19:40	JetBrains PyCharm	7 KB
Polishing_S2.py	2025/1/5 19:41	JetBrains PyCharm	11 KB
