# Supplementary material for: Adaptive force-position-speed collaborative process planning and roughness prediction for robotic polishing
Source: PLoS One. 2025 Sep 3;20(9):e0330979. doi: 10.1371/journal.pone.0330979 (PMC12407474; doi:10.1371/journal.pone.0330979)
Supplement: Attachment — Submitted filename: Review_PONE_D_25_00868_08.07.2025.pdf [file pone.0330979.s003.pdf]

Review

General Comments

The manuscript addresses a relevant topic and presents valuable contributions. However, there are several issues throughout the text that should be revised to improve the clarity, consistency, and scientific rigor of the work. The comments below include specific technical and editorial observations that, once addressed, will strengthen the overall quality of the manuscript. The most critical points relate to inconsistencies in notation, misuse of physical units, and lack of clarity in key equations. I encourage the authors to carefully consider each of the suggestions provided.

1. **Page 5, Equation (1):** Equation (1) indicates that the function $h(x, y)$ depends solely on the spatial variables x and y; however, the explicit expression shows a dependency on time t. This creates an inconsistency between the functional notation and the mathematical expression. It is recommended to revise whether the correct notation should be $h(x, y, t)$, which would be more appropriate if temporal dependency indeed exists.

2. **Page 7, Line 186:** The sentence reads, "*the positive polishing pressure was 20 N*". However, pressure should be expressed as force per unit area, i.e., in N/m^2 or Pascals (Pa). Expressing pressure as a scalar force can lead to conceptual misunderstandings. It is advised to correct the unit or clarify that a normal force was applied over a specific area.

3. **Page 10, Lines 262–263:** The phrase "*an imaginary spring-damper system*" is used, although Equation (16) includes a mass term m, indicating that the modeled system is in fact a mass-spring-damper system. Therefore, it would be more accurate to refer to it as such, rather than omitting the inertia component.

4. **Page 10, Line 272:** The relation between the measured value x_m, the estimated true value $\hat{x}$, and the error δx is written as:
$$x_m = \hat{x} - \delta x$$
However, this contradicts the standard definition of error, which is:
$$\delta x = x_m - \hat{x} \quad \Rightarrow \quad x_m = \hat{x} + \delta x$$
The expression in the text should be corrected to be consistent with the definition of error. The subsequent substitutions in the manuscript appear to follow the correct notation.

5. **Page 11, Equations (22) and (23):** It would be helpful to clarify whether, from a single preceding equation (21), it is valid to derive both the velocity error $\dot{e}$ and the acceleration error $\ddot{e} = \ddot{x}_d - \ddot{\hat{x}}_e$ as presented in Equations (22) and (23), respectively. Although mathematically possible, it is advisable to explain the derivation or the assumptions made in order to justify the separation into distinct expressions.

6. **Page 15, Line 385:** It is stated that the system operates with a constant sampling time of 0.01 seconds. However, platforms such as *xArm* include multiple layers of processing (e.g., safety mechanisms, internal verification in the VirtualBox, USB-serial communication), which can introduce non-negligible variability in sampling times. How is this variability addressed, and how is a constant sampling rate ensured in such a context? A discussion of this issue is recommended.

7. **Page 16, Line 408:** The feed speed v_f is presented in units of mm/min, while in Table 2, the same variable is expressed in m/s. It is recommended to standardize the units throughout the manuscript or at least provide an explicit conversion to facilitate interpretation.

8. **Page 22, Line 576:** The elastic strain is expressed in units of mm/mm. Although this is technically correct, it may lead to confusion, as strain is a dimensionless quantity. It is suggested to clarify that the value is unitless, since using mm/mm, cm/cm, or km/km would yield the same result.

9. **Page 23, Figures 16 and 17:** The axes in Figures 16 and 17 do not specify the units being used. It is recommended to include this information clearly in the captions or axis labels to enable proper quantitative interpretation of the results.
