# Supplementary material for: Adaptive force-position-speed collaborative process planning and roughness prediction for robotic polishing
Source: PLoS One. 2025 Sep 3;20(9):e0330979. doi: 10.1371/journal.pone.0330979 (PMC12407474; doi:10.1371/journal.pone.0330979)
Supplement: Attachment — Submitted filename: Response to Reviewers.docx [file pone.0330979.s002.docx]

**Reviewers' comments:**

**Reviewer 1**

1: The term RBF is used several times before being adequately defined. To improve the clarity of the text, it is recommended that the author define it on its first appearance.

Response:

Thank you for your helpful comment. We agree that the term “RBF” should be clearly defined upon its first appearance. Accordingly, we have revised the manuscript to define “RBF” as “Radial Basis Function” when it first appears in Section 2.3.2. The revised sentence reads: “A Radial Basis Function (RBF) neural network, a type of feedforward neural network using radial basis functions as activation functions, is employed to approximate unknown dynamics in the control model.”

2: The equations (Preston, impedance control, etc.) require greater clarity to facilitate understanding. For example, some terms, such as Fc and Fs, are not defined in the text, making their interpretation difficult. It is recommended that descriptions of the parameters used be included.

Response:

To improve clarity, we carefully reviewed all relevant equations (especially Sections 2.1 and 2.3) and added explicit definitions of key parameters such as Fn (normal force), p(x,y,t) (contact pressure), etc. In Section 2.3.2. Trajectory position control, all parameters of the LuGre friction model are explained. The LuGre model is a widely used approach for estimating and compensating for friction in robotic systems. It provides a detailed representation of friction dynamics by capturing both static and dynamic friction behaviors. The model describes friction as a combination of Coulomb friction, viscos friction, and a dynamic state variable representing the microscopic bristle deformation between contact surfaces. The LuGre friction model can be expressed as formula (31).

$\left\{ \begin{aligned} &f_{n}\left( \dot{q} \right)=\sigma_{0}z+\sigma_{1}\dot{z}+\sigma_{2}\dot{q} \\ &\dot{z}=\dot{q}-\sigma_{0}\frac{\left| \dot{q} \right|}{g(\dot{q})}z \\ &g\left( \dot{q} \right)=F_{c}+(F_{s}-F_{c})e^{-{(\dot{q}/v_{s})}^{2}} \end{aligned} \right.$	(31)

The values of the LuGre friction model parameters determined in this study, considering the kinematic parameters of the joints, are shown in the following table.

| Parameter Value | Unit | Description |
| --- | --- | --- |
| σ0 = 84000 | Nm/rad | Stiffness coefficient |
| Fs = 8.16 | Nm | Static friction torque |
| Fc = 3.82 | Nm | Coulomb friction torque |
| σ1 = 260 | Nm*s/rad | Damping coefficient |
| σ2 = 28 | Nm*s/rad | Viscous friction coefficients |
| Vs = 0.0125 | rad/s | Velocity |

The process of calculating the LuGre friction model in robot control is shown in the figure below.

By utilizing the LuGre model, the controller can accurately estimate and compensate for the friction forces, improving the overall precision and performance of the robotic system in trajectory tracking tasks. This improves the understandability of model formulations and parameter explanations throughout the manuscript.

3: The article does not provide sufficient information on the parameters and coefficients used in the model equations, such as those of the neural networks, impedance control, PD, or PI controller. It is recommended that the author include more information on parameter selection, experimental setup, and other related aspects.

Response:

There are many parameters and coefficients of the neural network, impedance control, PD or PI controller involved in the paper. A new section 2.3.4 Parameter setting of controller has been added to list many parameter values.

The impedance control parameters were selected based on previous robot polishing research and tuning experimental experience: m = 0.15 kg, b = 30 Ns/m and k = 500 N/m. The iterative update law for environmental deviation compensation adopted a system sampling period λ = 0.01s, a forward learning rate η= 0.05 and ς = 0.05, and a gradient reverse iteration calculation step size α = 0.001.

The proportional gain and integral gain used in the PI position controller were *K_P_* = **diag**(30, 30, 30, 20, 20, 20) and *K_I_* = **diag**(5, 5, 5, 3, 3, 3), respectively, which were determined by iterative adjustment to balance steady-state tracking and responsiveness.

The RBF neural network consists of 3 input nodes (joint position errors), 1 hidden layer with 10 radial basis neurons using Gaussian activation function, and 1 output node. The radial function expansion is set to 1.5. The learning rate for weight adaptation is set to 0.01, and the initial weights are randomly initialized from a uniform distribution in the range [-0.5, 0.5].

To estimate the friction terms $f_{n}\left( \dot{q} \right)$, the parameters of the LuGre friction model are selected as shown in Table 1.

4: The results section of the article is very short and lacks an analysis that helps to interpret the data more in-depth. A more complete discussion of the experimental findings is recommended.

Response:

We have significantly revised and expanded Sections 3.2-3.4 (Results and Discussion) in the revised manuscript to provide a more comprehensive interpretation of the experimental data and model performance.

(1) We have added a comparison between the original and proposed polishing force control strategies, emphasizing the smoothness of the response and the stability of the force. The updated text explains the behavior of the different polishing stages (non-contact, contact, and steady polishing) and emphasizes how the proposed impedance strategy reduces overshoot and improves tracking accuracy.

Compared with the original control method, the proposed impedance-based force control significantly reduces the overshoot and oscillation in steady-state polishing (region C), allowing the actual force to closely track the target value (22.5 N). This result validates the robustness and adaptability of the proposed controller.

(2) We enrich the comparison of polishing results by examining the average reduction in surface roughness between the original and proposed methods, and show a quantitative improvement: the proposed strategy reduces the surface roughness from 0.52947 µm to 0.41943 µm, an improvement of 20.79%. This indicates superior consistency and smoother surface finishes, which is particularly important for high-precision parts.

(3) The results of BPNN prediction and RSM optimization are further discussed, focusing on the interaction between factors: Response surface analysis shows that there is a synergistic effect between tool speed and polishing force, with medium and high values of both tending to produce the best roughness. Feed rate exhibits a decreasing effect, while sandpaper type exhibits a nonlinear effect. These insights help guide the selection of process parameters.

(4) Reasoning about structural safety and its relevance to force control design is added: The deformation of 0.0403 mm and the equivalent strain of 1.01×10⁻⁵ confirm the structural stability of the robot under working conditions. These findings validate the assumptions used in the design of the impedance controller and justify the applied polishing force.

(5) Explanation of the low RMSE value and generalization ability: The RMSE of 0.00011 demonstrates the high accuracy of the DBO-BPNN prediction model. The close values between the training and test sets reflect the generalization ability of the model, which enables it to reliably estimate surface roughness under unseen conditions.

5: The justification fails to fully demonstrate the study's relevance. The author is encouraged to include a more detailed discussion of the limitations of current techniques and why the proposed method is superior to other strategies.

Response:

To address this issue, we added the following discussion to the Introduction: Despite advances in robotic polishing, many existing methods still face limitations in adapting to variable surface curvature, maintaining consistent polishing force under dynamic conditions, and optimizing process parameters to achieve surface quality. Traditional force control strategies often suffer from overshoot and poor anti-interference capabilities, while fixed trajectory planning methods have difficulty coping with uneven material removal on complex surfaces. Therefore, this study integrates curvature-adaptive trajectory generation, a process planner based on improved dung beetle optimization, and an adaptive impedance control framework to overcome these challenges. The proposed approach combines intelligent prediction, real-time adaptation, and multi-factor optimization to provide a unified solution for high-precision robotic polishing.

Added a new discussion to the Conclusion: Compared with existing robotic polishing strategies, the proposed framework exhibits superior robustness and adaptability. The curvature-adaptive interpolation method ensures high-precision trajectory tracking on surfaces with complex geometries, overcoming the limitations of traditional equal-step or uniform parameter trajectory planners. The adaptive impedance control strategy can effectively mitigate transient force impacts and compensate for environmental uncertainties, thereby improving force stability and surpassing traditional fixed-gain methods. In addition, the DBO-BPNN model combines global optimization with predictive learning to achieve accurate surface roughness estimation under different process settings. These improvements make the framework highly suitable for industries with strict precision requirements such as aerospace, mold manufacturing, and optical component processing.

6: There is no justification for selecting the iterative PD controller over other options. An explanation of the rationale for this method is encouraged.

Response:

The original paper did not adequately explain the rationale for choosing an iterative PD controller. We now add rationales for the choice based on theoretical applicability, practical ease of operation, and compatibility with adaptive impedance control. Section 2.3.1, new paragraph: In this study, an iterative PD control approach was chosen to compensate for deformation deviations of the environment in real time. Compared with model-based control approaches such as adaptive backstepping or sliding mode control, the iterative PD approach strikes a good balance between computational simplicity and adaptability. It does not require an accurate dynamic model of the workpiece or contact surface and is particularly effective in practical robotic applications where the environment stiffness varies and there are uncertainties. In addition, its integration with the impedance control structure allows the system stiffness to be dynamically adjusted using a gradient descent method, thereby improving transient force control performance without causing instabilities.

**Reviewer 2**

1: While the problem is well stated, the manuscript requires significant revisions before it can be considered for publication. There are several mathematical and theoretical issues that need to be addressed. For instance, in equation (3), the feed velocity appears in the denominator, which could lead to ill-posed conditions if it approaches zero. Additionally, there is a sign error in equation (25).

Response:

This critical mathematical problem does need to be considered. During the revision, we consulted various literatures related to Preston's equation and finally determined the modification scheme. Formula (3) in the manuscript was modified as follows:

$v\left( x,y,t \right)=\sqrt{v_{t}^{2}\left( x,y,t \right)+v_{f}^{2}\left( x,y,t \right)}$	(3)

This formula is based on the assumption that the tangential velocity of the rotating polishing tool and the feed rate of the robot are orthogonal components. It is consistent with the traditional modeling in polishing dynamics and avoids any division of small values, thus ensuring numerical stability. The feed rate of all experiments was kept above the minimum threshold (5 mm/min) to ensure effective material removal and prevent degradation.

We thank the reviewer again for pointing out the unclear gradient formulation in Equation (25). The change in stiffness over time is tracked using the gradient descent method. The gradient of the optimization objective equation is calculated as shown in equation (25). Here, $f_{0}$ represents the stiffness coefficient in the impedance model. It directly affects the system's compliance during contact. The gradient $\nabla J$ expresses the sensitivity of the force error for this stiffness, allowing adaptive adjustment to minimize 𝐽(𝑡). Here, it is assumed that the effect of $\dot{e_{f}}$ on $f_{0}$ is very small (the second-order effect can be approximately ignored).

$\begin{matrix} \nabla J(f_{0})=\mu\cdot e_{f}\cdot\frac{\partial e_{f}}{\partial f_{0}}+\nu\cdot\dot{e_{f}}\cdot\frac{\partial\dot{e}_{f}}{\partial f_{0}} \\ \nabla J\left( f_{0} \right)\approx-\mu\cdot e_{f}\cdot(x_{d}-x) \end{matrix}$	(25)

In order to minimize the contact force error and trajectory position error of the robot end effector, the change in stiffness $f_{0}$ changes in the opposite direction of the gradient as shown in formula (26), where $\alpha$ is the iterative calculation step size, $0<\alpha<1$.

$f_{0}\left( t \right)=f_{0}\left( t-\lambda\right)-\alpha\nabla J(f_{0})$	(26)

2: Beyond these, there are also conceptual concerns. For example, the cost function in equation (24) is presented as minimizing position error, yet it only depends on velocity and force errors. In the Lyapunov analysis, equation (38) does not hold as stated. In equation (39), the authors introduce a small constant C, but no proof is provided for its existence. Furthermore, the claim of asymptotic stability relies on Barbalat’s lemma without a sufficiently rigorous justification, as the lemma does not typically lead to such a conclusion.

Response:

The generalized tracking error $r=\dot{e}+\Lambda e$ includes both velocity and position errors. Therefore, minimizing *r^2^* also indirectly minimizes position tracking error eee.

An optimization control strategy is established with the control objectives of minimizing the contact force error, velocity tracking error, and trajectory position tracking error, and the optimization objective equation is selected as (24).

$J\left( t \right)=\frac{1}{2}\left( \mu{e_{f}}^{2}+v_{1}\dot{e}^{2}+v_{2}e^{2} \right)$	(24)

The change in stiffness over time is tracked using the gradient descent method. The gradient of the optimization objective equation is calculated as shown in equation (25). Here, $f_{0}$ represents the stiffness coefficient in the impedance model. It directly affects the system's compliance during contact. The gradient $\nabla J$ expresses the sensitivity of the force error for this stiffness, allowing adaptive adjustment to minimize 𝐽(𝑡). Here, it is assumed that the effect of $\dot{e_{f}}$ on $f_{0}$ is very small (the second-order effect can be approximately ignored). The third term in the gradient expression (25) is neglected due to its minimal sensitivity and weak coupling with the stiffness parameter.

$\begin{matrix} \nabla J=\frac{dJ}{df_{0}}=\mu\cdot e_{f}\cdot\frac{\partial e_{f}}{\partial f_{0}}+\nu_{1}\cdot\dot{e}\cdot\frac{\partial\dot{e}}{\partial f_{0}}+v_{2}\cdot e\cdot\frac{de}{df_{0}} \\ \nabla J\approx-\mu\cdot e_{f}\cdot(x_{d}-x) \end{matrix}$	(25)

In order to minimize the contact force error and trajectory position error of the robot end effector, the change in stiffness $f_{0}$ changes in the opposite direction of the gradient as shown in formula (26), where $\alpha$ is the iterative calculation step size, $0<\alpha<1$.

$f_{0}\left( t \right)=f_{0}\left( t-\lambda\right)-\alpha\nabla J$	(26)

To address this, we have revised Equation (38) to clearly reflect the impact of the RBF approximation error.

$\dot{V}=-r^{T}K_{p}r-K_{r}\left\| r \right\|_{1}+\epsilon$	(38)

Where, $\epsilon$ denotes the approximation error of the RBF neural network, $\left\| r \right\|_{1}$ represents the L1 norm of vector 𝑟, also known as the absolute value norm. In robot control, by properly choosing the values of *Kp>0* and *Kr>0*, we can ensure that the approximation error *ϵ* of the RBF neural network is bounded. According to the Universal Approximation Theorem, there exists a constant $C>0$ such that $|\epsilon|<C$. Thus, we obtain the formula (39).

$\dot{V}=-\left\| r \right\|^{2}-K_{r}\left\| r \right\|_{1}+C$	(39)

Where, *C* is a small constant representing the approximation error of the RBF neural network. By choosing sufficiently large control gains *Kp>0* and *Kr>0*, we ensure that the negative terms dominate *C*, i.e., $\left\| r \right\|^{2}+K_{r}\left\| r \right\|_{1}>C$.

Therefore, $\dot{V}<0$, and the Lyapunov function *V*(*t*) is strictly decreasing. According to LaSalle’s Invariance Principle, all trajectories converge to the largest invariant set where $\dot{V}=0$, which implies $r\to0$ as $t\to\infty$. Thus, the system is asymptotically stable.

3: In addition to these technical issues, the manuscript would benefit from improvements in clarity and presentation. The quality of the images is low, and there are several typographical and grammatical errors that affect readability. For instance, in the abstract, the statement that the prediction model reaches 0.0001 lacks context and units, and the reported improvement of 20.79% is presented without sufficient explanation.

Response:

We have carefully addressed each issue as follows:

(1) All figures in the manuscript have been replaced with high-resolution versions (300 dpi or higher). Line charts, experimental diagrams, and comparison plots (e.g., Figures 13, 20, and 21) have been exported in vector format to ensure clarity. In addition, legends, labels, and units have been enhanced to improve readability.

(2) We have revised the abstract to clarify the context and units of the performance metrics. The root mean square error (RMSE) is now reported with units, and the meaning of the 20.79% improvement has been explained. The revised sentences now read:

The proposed DBO-BPNN model achieved a root mean square error (RMSE) of 0.0001 μm in surface roughness prediction, demonstrating high accuracy. In addition, the surface roughness of the polished workpieces was reduced by 20.79% on average compared to the baseline method.

(3) We have thoroughly revised the manuscript to correct typographical and grammatical issues. All sections have been checked using proofreading tools and carefully reviewed for consistent technical terminology. This improves the overall readability and presentation quality of the paper.

4: Finally, the manuscript introduces multiple methodologies, including Dug Beetle Optimization, Back Propagation Neural Networks (misused term), Radial Basis Functions, Finite Element Analysis, and Adaptive Impedance Control. However, a clearer methodological framework is needed to demonstrate how these tools are integrated in a coherent and structured manner.

Response:

This study integrates multiple algorithmic modules to address different aspects of robotic polishing, all of which have been updated in Figure 1.

DBO is used to optimize polishing parameters (force, speed, tool type); BPNN is trained based on experimental data to predict surface roughness under different settings; RBF neural network enhances adaptive impedance control by approximating unknown dynamic terms; Finite element analysis verifies structural deformation and verifies physical feasibility; All modules are input into the adaptive control framework to ensure stable polishing and high surface quality.

Fig. 1 Overall workflow of this study

We have corrected and standardized the terminology. Specifically, we now refer to the neural network used for surface prediction as a back-propagation based feed-forward neural network (BPNN) and the control-related model as an RBF neural network used for dynamic approximation. Each revision in the text has been made to follow these standardized terms. Please review.
