# Supplementary material for: Adaptive force-position-speed collaborative process planning and roughness prediction for robotic polishing
Source: PLoS One. 2025 Sep 3;20(9):e0330979. doi: 10.1371/journal.pone.0330979 (PMC12407474; doi:10.1371/journal.pone.0330979)
Supplement: Attachment — Submitted filename: Response_to_Reviewers_auresp_2.docx [file pone.0330979.s004.docx]

**Reviewers' comments:**

**Reviewer 1**

1: **Page 5, Equation (1):** Equation (1) indicates that the function *h*(*x, y*) depends solely on the spatial variables *x* and *y*; however, the explicit expression shows a dependency on time *t*. This creates an inconsistency between the functional notation and the mathematical expression. It is recommended to revise whether the correct notation should be *h*(*x, y, t*), which would be more appropriate if temporal dependency indeed exists.

Response:

We have corrected the notation in Equation (1) from h(x,y) to h(x,y,t) to accurately reflect the time-dependent nature of material removal during the polishing process.”

2: **Page 7, Line 186:** The sentence reads, “*the positive polishing pressure was 20 N* ”. However, pressure should be expressed as force per unit area, i.e., in N/m2 or Pascals (Pa). Expressing pressure as a scalar force can lead to conceptual misunderstandings. It is advised to correct the unit or clarify that a normal force was applied over a specific area.

Response:

We have revised the statement to “a normal contact force of 20 N was applied,” clarifying that it is the force component, and the corresponding pressure is determined based on the contact area as modeled in the material removal section. The corresponding contact pressure in this study was approximated by the circular area of the polishing tool.

3: **Page 10, Lines 262–263:** The phrase “*an imaginary spring-damper system*” is used, although Equation (16) includes a mass term *m*, indicating that the modeled system is in fact a mass spring-damper system. Therefore, it would be more accurate to refer to it as such, rather than omitting the inertia component.

Response:

We agree with the reviewer’s suggestion and have corrected the description to “mass-spring-damper system” to better reflect the inclusion of inertia in Equation (16).

4: **Page 10, Line 272:** The relation between the measured value *xm*, the estimated true value ˆ*x*, and the error *δx* is written as: *xm* = ˆ*x − δx* However, this contradicts the standard definition of error, which is: *δx* = *xm − x*ˆ *⇒ xm* = ˆ*x* + *δx* The expression in the text should be corrected to be consistent with the definition of error. The subsequent substitutions in the manuscript appear to follow the correct notation..

Response:

Thank you for identifying this inconsistency. We have corrected the expression to follow the standard definition of error: xm=x^+δx, and ensured that all related substitutions are consistent throughout the manuscript.

5: **Page 11, Equations (22) and (23):** It would be helpful to clarify whether, from a single preceding equation (21), it is valid to derive both the velocity error ˙*e* and the acceleration error *e*¨ = ¨*xd − x*¨ˆ*e* as presented in Equations (22) and (23), respectively. Although mathematically possible, it is advisable to explain the derivation or the assumptions made in order to justify the separation into distinct expressions.

Response:

We have now added explanatory text preceding Equations (22) and (23) to clarify that these expressions are derived under the assumption of continuous differentiability of the impedance error function. To further evaluate the dynamic behavior of the impedance control model, the position error term e is differentiated with respect to time to derive the velocity and acceleration errors. Assuming that all relevant variables can be differentiated with respect to time, the first-order derivative of the position error is e ̇, and the second-order derivative is e ̈=x ̈_d-x ̂ ̈_e. This allows the calculation of formulas (22) and (23).

6: **Page 15, Line 385:** It is stated that the system operates with a constant sampling time

of 0.01 seconds. However, platforms such as *xArm* include multiple layers of processing (e.g., safety mechanisms, internal verification in the VirtualBox, USB-serial communication), which can introduce non-negligible variability in sampling times. How is this variability addressed, and how is a constant sampling rate ensured in such a context? A discussion of this issue is recommended..

Response:

The xArm platform may introduce timing variability due to internal safety protocols and USB-serial communication delays. In our implementation, we measured the actual update intervals using timestamped logs and verified that the effective sampling interval remained consistently close to 0.01 s, with variations within ±1 ms. This quasi-periodic sampling was deemed acceptable for the stability of the proposed control algorithm. We have added a clarification in Section 2.3.4 to address this point.

7: **Page 16, Line 408:** The feed speed *vf* is presented in units of mm/min, while in Table 2, the same variable is expressed in m/s. It is recommended to standardize the units throughout the manuscript or at least provide an explicit conversion to facilitate interpretation.

Response:

Thank you for highlighting this inconsistency. We have standardized the unit of feed speed vf to mm/min throughout the manuscript and updated Table 2 accordingly to maintain consistency and clarity.

8: **Page 22, Line 576:** The elastic strain is expressed in units of mm/mm. Although this is technically correct, it may lead to confusion, as strain is a dimensionless quantity. It is suggested to clarify that the value is unitless, since using mm/mm, cm/cm, or km/km would yield the same result.

Response:

Strain is a dimensionless quantity. While the value was initially expressed as mm/mm for illustrative clarity, we have revised the manuscript to indicate that equivalent elastic strain is fundamentally unitless, and have updated the related sentence accordingly. The maximum equivalent elastic strain is 1.0117×10⁻⁵, which is unitless.

9: **Page 23, Figures 16 and 17:** The axes in Figures 16 and 17 do not specify the units being used. It is recommended to include this information clearly in the captions or axis labels to enable proper quantitative interpretation of the results.

Response:

We have updated the captions of Figs 16 and 17 to include the units of the axes (in millimeters) to ensure clear quantitative interpretation.
